# First Complete Genome Sequence of Human Coronavirus HKU1 from a Nonill Bat Guano Miner in Thailand

**DOI:** 10.1128/MRA.01457-18

**Published:** 2019-02-07

**Authors:** Yutthana Joyjinda, Apaporn Rodpan, Pongtorn Chartpituck, Krairerk Suthum, Suphaluk Yaemsakul, Thaniwan Cheun-Arom, Saowalak Bunprakob, Kevin J. Olival, Martha M. Stokes, Thiravat Hemachudha, Supaporn Wacharapluesadee

**Affiliations:** a Thai Red Cross Emerging Infectious Disease-Health Science Centre, World Health Organization Collaborating Centre for Research and Training on Viral Zoonoses, Chulalongkorn Hospital, Faculty of Medicine, Chulalongkorn University, Bangkok, Thailand; b The 5^th^ Office of Disease Prevention and Control, Ratchaburi, Thailand; c Department of Biology, Faculty of Science, Ramkhamhaeng University, Bangkok, Thailand; d EcoHealth Alliance, New York, New York, USA; e Cooperative Biological Engagement Program, Defense Threat Reduction Agency (DTRA), Washington, DC, USA; University of California, Riverside

## Abstract

Human coronavirus HKU1 (HCoV-HKU1) was first detected in a patient with viral pneumonia from Hong Kong in 2004. Here, we report the first complete genome sequence of HCoV-HKU1 from Thailand, obtained from a nonill person who worked in a bat cave. Phylogenetic tree analysis revealed it as a group B HCoV-HKU1.

## Abstract (precis)

Human coronavirus HKU1 (HCoV-HKU1) was first detected in a patient with viral pneumonia from Hong Kong in 2004. Here, we report the first complete genome sequence of HCoV-HKU1 from Thailand, obtained from a nonill person who worked in a bat cave.

## ANNOUNCEMENT

There are four species of endemic human coronavirus (HCoV) currently recognized by the International Committee for the Taxonomy of Viruses, namely, HCoV-OC43, -229E, -NL63, and -HKU1, and two epidemic CoVs, including severe acute respiratory syndrome (SARS)-CoV and the Middle East respiratory syndrome (MERS)-CoV discovered in 2003 and 2012, respectively (1). Bats are believed to be the ancestral hosts of alpha- and beta-CoV, including SARS-CoV and MERS-CoV (2, 3). A group C betacoronavirus (MERS-related CoV) was detected from dry bat guano collected from a cave in Thailand where bat guano is sold for use as fertilizer (4). Nasopharyngeal swabs of bat guano miners (*n* = 34) in Ratchaburi Province, Thailand, were collected and sent to the Thai Red Cross-Emerging Infectious Diseases laboratory to test for CoVs using conventional PCR targeting the betacoronavirus RNA-dependent RNA polymerase (RdRp) gene (5). One sample was positive for the coronavirus HKU1 strain, and its whole genome was sequenced by using next-generation sequencing (NGS).

The nasopharyngeal swab found CoV positive by conventional PCR was subjected to metagenomic sequencing on the Illumina MiSeq platform. RNA was extracted using a QIAamp viral RNA mini kit (Qiagen, Germany), followed by DNase treatment. A DNA library was prepared using a TruSeq total RNA with Ribo-zero globin kit (Illumina). The quality and quantity of the DNA library was estimated by using the QIAxcel Advanced system and QIASeq library quantification kit, respectively (Qiagen, Germany). The 17 pmol of DNA library was injected into the flow cell and sequenced using a MiSeq reagent kit version 3.

A total of 59,704,490 sequencing reads from 2 × 150-bp paired ends which, passing a quality score of 30, was used for data analysis. Host DNA (human genome from GATK resource bundle) removal was done using the SNAP (version 0.15.4) alignment tool (6). Mapping assembly was performed using HKU1 (GenBank accession number AY884001) as the reference strain by using the Burrows-Wheeler Aligner (BWA) program (7). A total of 6,475,827 reads mapped to HCoV-HKU1 at a depth of 29,000×, which covered the whole genome of 29,811 bp. The consensus sequence of HCoV-HKU1 with a G+C content of 65% was generated using SAMtools mpileup (8). This is the first complete genome of HCoV-HKU1 isolated from Thailand.

**FIG 1:**
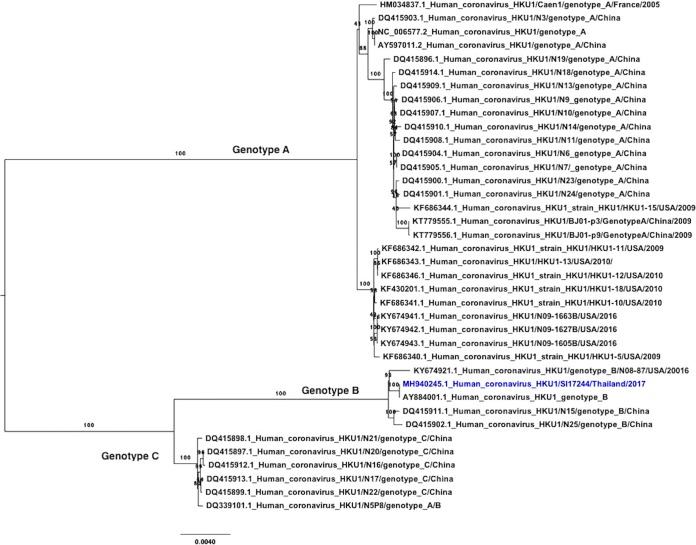
Phylogenetic tree of HCoV-HKU1 using maximum likelihood method with 1,000 bootstrap replicates. The whole-genome sequence of HCoV-HKU1 (29,811 bp) from Thailand is colored in blue; it belonged to group B genotype and was closely related to HCoV-HKU1 from Hong Kong (GenBank accession number AY884001).

Phylogenetic analysis of our HCoV-HKU1 whole-genome and reference sequences from the GenBank database, using maximum likelihood algorithm on the MEGA6 program, showed that our sequence is in the same clade with sequences from China and the United States (Fig. 1). It belonged to the group B genotype and was closely related to HCoV-HKU1 from Hong Kong (GenBank accession number AY884001) that was isolated in 2006 (99% nucleotide identity) (9). It is worth noting that this virus was detected from an individual with a high level of occupational exposure to bat feces and, thus, an elevated level of zoonotic virus spillover. While this individual was likely exposed to HCoV-HKU1 due to person-to-person transmission and not via exposure from bats, our surveillance strategy and viral characterization pipeline provide valuable insight into the circulation of endemic infectious diseases in Thailand and increase the country’s preparedness for other novel emerging infectious diseases.

### Data availability.

The human coronavirus HKU1 strain reported here was deposited in GenBank under the accession number MH940245 and SRA accession number PRJNA509533.
